# NAAG peptidase inhibition in the periaqueductal gray and rostral ventromedial medulla reduces flinching in the formalin model of inflammation

**DOI:** 10.1186/1744-8069-8-67

**Published:** 2012-09-12

**Authors:** Toshihiko Yamada, Daiying Zuo, Tatsuo Yamamoto, Rafal T Olszewski, Tomasz Bzdega, John R Moffett, Joseph H Neale

**Affiliations:** 1 Department of Anesthesiology, Kumamoto University, Kumamoto, Japan; 2 Department of Biology, Georgetown University, Washington, DC, USA; 3 Department of Pharmacology, Shenyang Pharmaceutical University, Shenyang, China; 4 Department of Anatomy, Physiology and Genetics, Neuroscience Program, Uniformed Services University of the Health Sciences, Bethesda, MD, USA; 5 Department of Biology, Georgetown University, 37th and O Sts., NW, Washington, DC 20057, USA

**Keywords:** Analgesia, NAAG, PAG, RVM, mGluR3, LY341495, Inflammatory pain, Microdialysis

## Abstract

**Background:**

Metabotropic glutamate receptors (mGluRs) have been identified as significant analgesic targets. Systemic treatments with inhibitors of the enzymes that inactivate the peptide transmitter N-acetylaspartylglutamate (NAAG), an mGluR3 agonist, have an analgesia-like effect in rat models of inflammatory and neuropathic pain. The goal of this study was to begin defining locations within the central pain pathway at which NAAG activation of its receptor mediates this effect.

**Results:**

NAAG immunoreactivity was found in neurons in two brain regions that mediate nociceptive processing, the periaqueductal gray (PAG) and the rostral ventromedial medulla (RVM). Microinjection of the NAAG peptidase inhibitor ZJ43 into the PAG contralateral, but not ipsilateral, to the formalin injected footpad reduced the rapid and slow phases of the nociceptive response in a dose-dependent manner. ZJ43 injected into the RVM also reduced the rapid and slow phase of the response. The group II mGluR antagonist LY341495 blocked these effects of ZJ43 on the PAG and RVM. NAAG peptidase inhibition in the PAG and RVM did not affect the thermal withdrawal response in the hot plate test. Footpad inflammation also induced a significant increase in glutamate release in the PAG. Systemic injection of ZJ43 increased NAAG levels in the PAG and RVM and blocked the inflammation-induced increase in glutamate release in the PAG.

**Conclusion:**

These data demonstrate a behavioral and neurochemical role for NAAG in the PAG and RVM in regulating the spinal motor response to inflammation and that NAAG peptidase inhibition has potential as an approach to treating inflammatory pain via either the ascending (PAG) and/or the descending pain pathways (PAG and RVM) that warrants further study.

## Background

Each of the current analgesic therapies has limited efficacy in treating inflammatory and neuropathic pain and some have significant side effects. As a result, there is a need to develop drugs with different targets in the nociceptive processing pathway. The heterotropic group II metabotropic glutamate receptor (mGluR2 and mGluR3) agonists have shown analgesic efficacy in animal models of inflammatory and neuropathic pain [1]. Consistent with this, activation of the type 3 metabotropic “glutamate” receptor (mGluR3) by the peptide neurotransmitter N-acetylaspartylglutamate (NAAG) reduces the flinching response to peripheral inflammation, reduces hyperalgesia induced by peripheral neuropathy and moderates the pain response in a model of bone cancer [2-9].

NAAG is one of the most prevalent transmitters in the mammalian nervous system [10]. It activates presynaptic mGluR3 receptors resulting in reductions in cAMP and cGMP levels [11-15], reduction in depolarization-induced calcium influx and inhibition of transmitter release [16-20]. The enzymes that inactivate synaptically released NAAG have been cloned [21-25] and potent inhibitors of these enzymes have been developed [26,27]. Systemic administration of these inhibitors, including ZJ43, reduces pain responses in animal models [2,4-9,28,29].

This research aimed to provide further insight into the mechanism of NAAG’s action in the pain processing pathway. We directly tested the hypothesis that the analgesic-like effects of NAAG peptidase inhibition in the formalin model of inflammation are mediated centrally in two well-recognized regions of the ascending and descending pain pathways, the periaqueductal gray (PAG) and the rostral ventromedial medulla (RVM). In parallel, the efficacy of systemically administered ZJ43 in elevating extracellular NAAG levels was assessed in these brain regions as was the influence of NAAG on the levels of inflammation-induced glutamate release.

## Results

**Figure 1:**
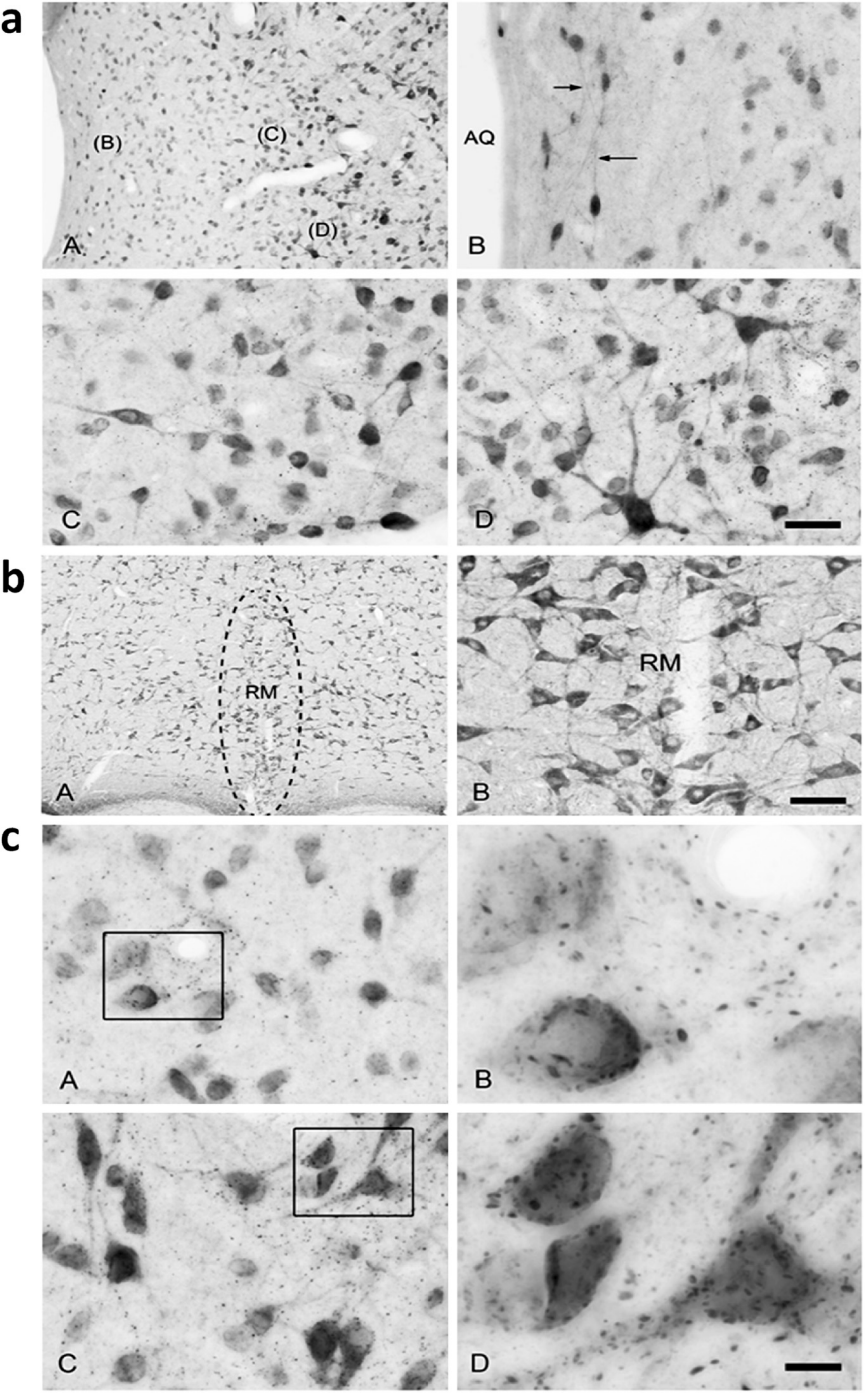
**a: NAAG immunoreactivity in neurons and processes of the lateral periaqueductal gray (PAG).** Regions denoted with letters in panel **A** show the areas of enlargement found in panels **B** – **D**. NAAG staining was relatively light in the region immediately surrounding the cerebral aqueduct (AQ), which contained small NAAG stained neurons and fine immunoreactive fibers (arrows in **B**). More neurons and processes were strongly immunoreactive for NAAG at the periphery of the PAG (**C** and **D**). The finely punctate staining represents high concentrations of NAAG as would be found in synaptic endings. Bar = 15 μm **B** – **D**. **b**: NAAG immunoreactivity in the raphe magnus (RM) of the brainstem (**A**). NAAG staining in the RM was similar to adjacent reticular areas with numerous moderately to strongly immunoreactive neurons. Many neuronal processes were immunoreactive for NAAG in the RM (**B**). Bar = 30 μm in B. **c**: NAAG-immunoreactivity in apparent synaptic contacts on neurons in the lateral PAG. Areas within boxes in **A** and **C** are enlarged in **B** and **D**. Panel **A** is from a region immediately adjacent to the cerebral aqueduct and Panel **C** is slightly further from the aqueduct. Apparent NAAG-containing synaptic contacts were observed on the surface of perikarya, major dendrites, and in the neuropil. Images acquired with extended depth of field. Bar = 5 μm **B** and **D.**

Using highly specific, multi-stage affinity purified antibodies (see Methods), NAAG immunoreactivity was observed in neurons and axons in the rat PAG and RVM (Figure 1a,b). Consistent with the potential of this transmitter to regulate neurotransmission via activation of presynaptic receptors in these brain regions, putative NAAGergic synapses were observed on the cell bodies and dendrites of PAG neurons, as well as in the neuropil (Figure 1c). While these data alone are insufficient to confirm the synaptic localization of this immunoreactivity, studies with this antibody at the ultrastructural level previously localized NAAG immunoreactivity to synaptic vesicles [30] and optic nerve transection studies demonstrated that NAAG immunoreactivity is associated with NAAG-containing synaptic terminals in various regions of the CNS [31,32]. Additionally, depolarization-induced calcium-dependent NAAG release has been demonstrated repeatedly [10,33]. These data on the presence of NAAG in the PAG and RVM provided a rationale for microinjection of the NAAG peptidase inhibitor ZJ43 into these regions of the pain transmission pathway.

**Figure 2:**
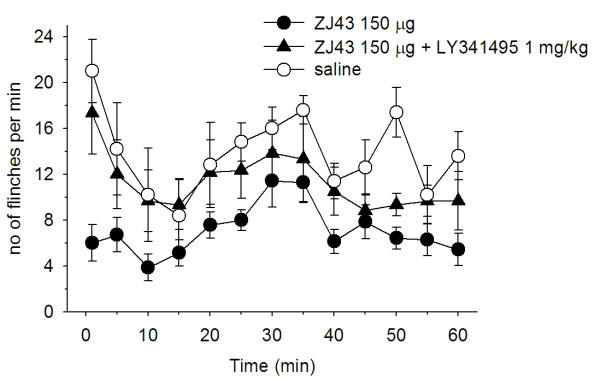
**Effect of microinjection of ZJ43 into the PAG.** Saline or ZJ43 or ZJ43 with LY341495 were microinjected into the right PAG 10 min prior to formalin injection into the dorsal surface of the contralateral footpad (0 time). Formalin injection induced two phases of hindpaw flinching in the saline injected control rats (n = 6). Microinjection of 150 μg of ZJ43 (n = 7) reduced both phases of the formalin-induced pain response. Pre-treatment with 1 mg/kg of the group II mGluR antagonist LY341495 (i.p.) (n = 6) reversed the effect of ZJ43 on the rapid and slow responses. Ordinate: number of flinches per min; abscissa: time after drug administration (min). Data in this and subsequent figures are presented as the mean +/− S.E.M.

**Figure 3:**
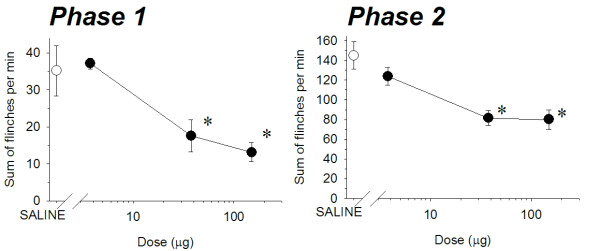
**ZJ43 dose response in the PAG.** Dose–response curves for ZJ43 injection into the PAG contralateral to the formalin injected site presenting the cumulative instances of formalin-evoked flinches during phase 1 and phase 2 of the formalin test (closed circles). Open circle represents sum of flinches by saline treated rats. ZJ43 or saline was microinjected into PAG 10 min before the formalin injection. Each point represents the mean of five to seven rats. ZJ43 reduced phase 1 and phase 2 flinching behavior 37.5 μg and 150 μg doses. * p < 0.05 as compared with the phase 1 or phase 2 responses in the saline treated rats (open circle).

**Figure 4:**
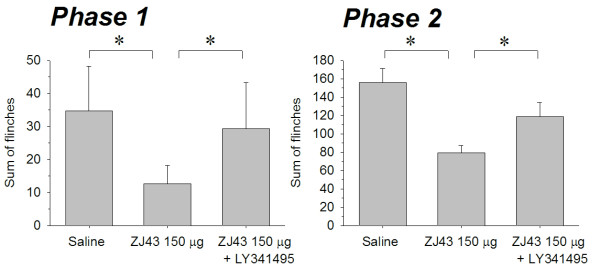
**Sum of flinches following microinjection of saline, ZJ43 or ZJ43 with LY341495 into the PAG.** Phase one data are the sum of flinches for the first 10 min. Phase two data are the sum of flinches from 10–60 min. Data are calculated from Figure 2. * p < 0.05.

**Figure 5:**
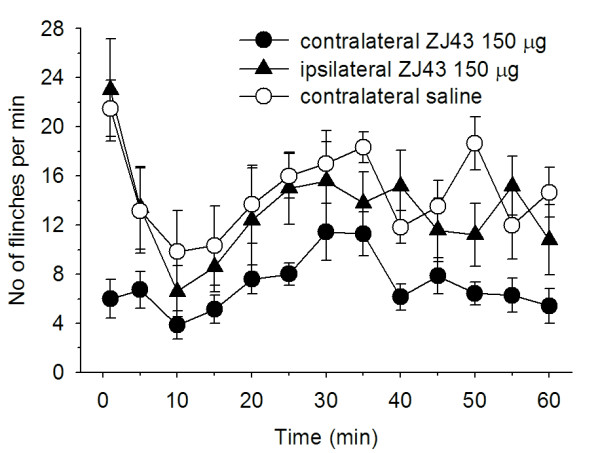
**ZJ43 microinjected into the PAG ipsilateral to the inflamed footpad.** Comparison of ZJ43 microinjection into the PAG contralateral and ipsilateral to the inflamed footpad. Ipsilateral data, n = 6. The contralateral ZJ43 and saline data are from Figure 2.

Subcutaneous injection of formalin in the rat footpad produced a reliable biphasic display of flinching of the injected paw (Figure 2) consistent with our previous studies [6-8]. Microinjection of ZJ43 (150 μg) into the PAG contralateral to the inflamed footpad significantly decreased the number of flinches in both the early and late phase. These effects of ZJ43 were dose-dependent (Figure 3) and blocked by i.p. injection with the mGluR2/3 receptor antagonist LY341495 (Figure 4, p < 0.05). We previously found that systemic injection of this antagonist alone had no significant effect on the response in the formalin or sciatic nerve ligation tests although a ceiling affect in the former and floor effect in the latter could have precluded detecting a change in response [6-8,29]. To confirm the restricted distribution of the peptidase inhibitor from the injection site, particularly its close proximity to the aqueduct, ZJ43 was injected into the PAG ipsilateral to injected footpad. The ipsilateral injection response was significantly different from the response following contralateral PAG injection (Figure 5, Phase 1: ipsilateral, 36.4 ± 6.5; contralateral, 12.7 ± 2.1, p < 0.005; Phase 2: ipsilateral, 136 ± 21.5; contralateral, 79.4 ± 7.8, p < 0.05) and not significantly different from the response following saline injection into the contralateral PAG (Phase 1: 34.7 ± 5.5; Phase 2: 156 ± 15.6).

**Figure 6:**
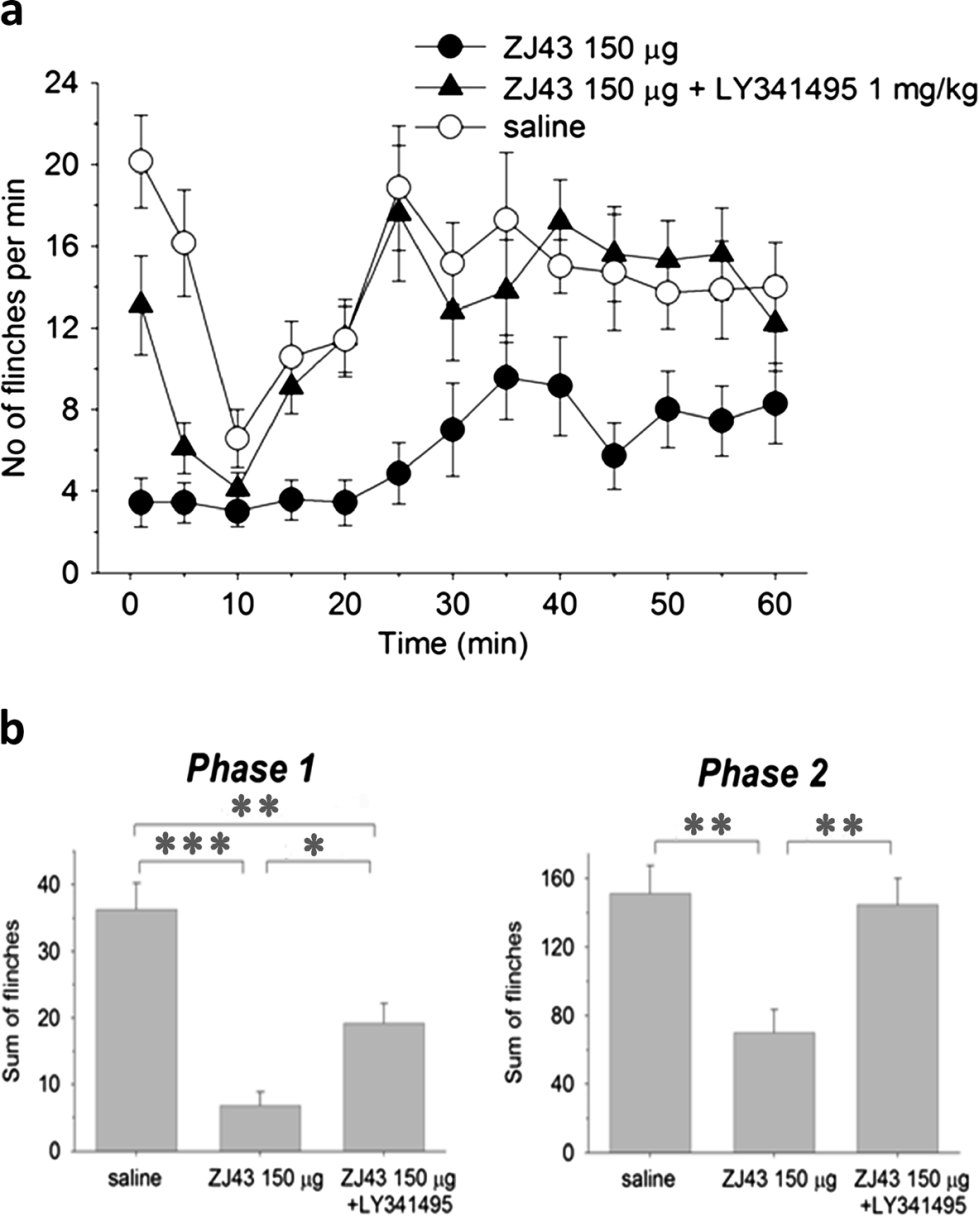
**ZJ43 microinjection into the RVM.** Formalin injection into the dorsal surface of the left rat hind-paw induced two phases of hindpaw flinching in the saline injected control rats (n = 7). **a**. Microinjection of 150 μg of ZJ43 (n = 7) into the contralateral RVM reduced both of phases of the pain response observed after the formalin injection. Pre-treatment with 1 mg/kg (i.p.) of the group II mGluR antagonist LY341495 (n = 10) blocked the effect of ZJ43 in the RVM. **b**. Sum of flinches following microinjection of saline, ZJ43 or ZJ43 with LY341495 into the RVM. Phase one data are the sum of flinches for the first 10 min. Phase two data are the sums of flinches from 10–60 min. Data are calculated from Figure 6a.

**Figure 7:**
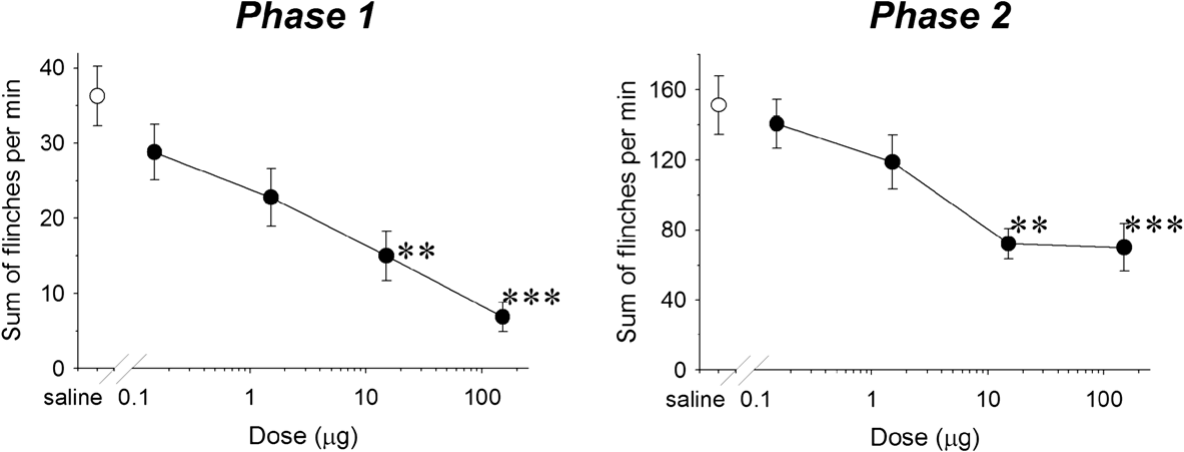
**Dose–response curves for ZJ43 injection into the midline RVM presenting the cumulative instances of formalin evoked flinches during phase 1 and phase 2 of the formalin test.** Drugs were administered into RVM 10 minutes before the formalin induction of inflammation. Each point represents the mean of responses of 5–7 rats. ZJ43 reduced phase 1 and phase 2 flinching behavior in a dose-dependent manner. **p < 0.01; ***p < 0.001 compared with the phase 1 or phase 2 responses in the saline treated rats.

NAAG-like immunoreactivity was found in the RVM, including the nucleus raphe magnus (Figure 1b). Microinjection of ZJ43 (150 μg) into the RVM also significantly reduced flinching in the rapid (p < 0.001) and slow (p < 0.01) phases of the inflammatory pain response (Figure 6a, b). The mGluR 2/3 antagonist LY341495 (i.p.) again reduced the effect of the peptidase inhibitor on the first phase of the flinching response (p < 0.05) and blocked the effect in the slow phase (p < 0.01) (Figures 6a, b). ZJ43 was effective in a dose-dependent manner (p <0.05) in both phases of the flinching response (Figure 7).

**Figure 8:**
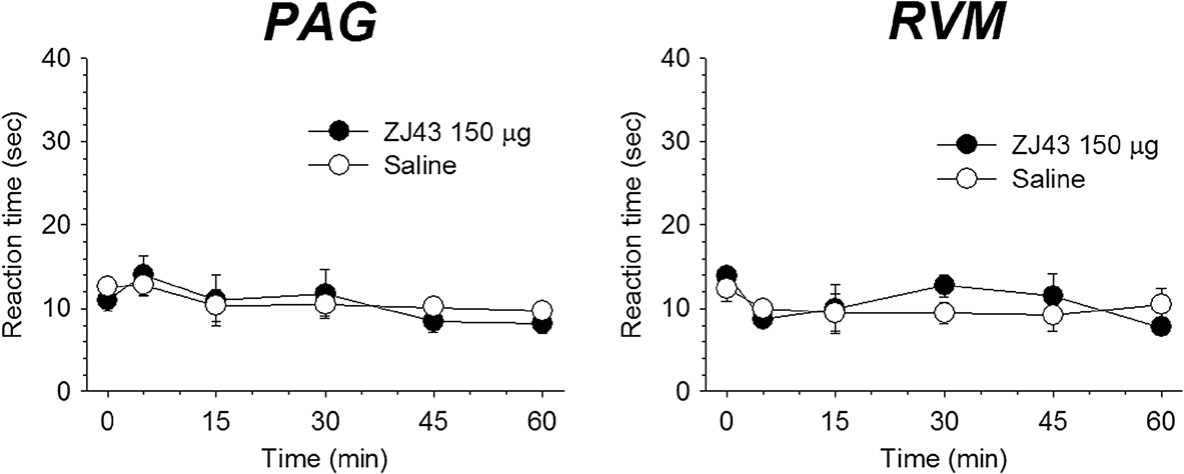
**Reaction times of rats placed on a 52.5°C surface following injection of NAAG peptidase inhibitor into the PAG and RVM.** Baseline latencies (O time) were established from 3 pretrial tests per animal and reaction times were assessed at different intervals after administration of saline or 150 μg ZJ43 into the PAG (n = 4, 6) and RVM (n = 4, 4).

Consistent with our previous reports that systemically administered ZJ43 and 2-PMPA failed to affect the thermal response latency [3,4], microinjection of ZJ43 (150 μg) into the PAG or RVM had no effect on the response in the hot plate test (Figure 8).

Our model of the efficacy of NAAG peptidase inhibition [3,33] predicts that ZJ43 will increase the extracellular levels of the peptide that in turn will activate presynaptic mGluR3 receptors to reduce release of glutamate or other transmitters. To test this, ZJ43 (150 mg/kg) was injected (i.p.) 15 minutes prior to footpad inflammation. This i.p. dose previously has been shown to reduce both the rapid and slow phase of the flinching response [6]. NAAG and glutamate levels were assessed by microdialysis in the PAG and RVM.

**Figure 9:**
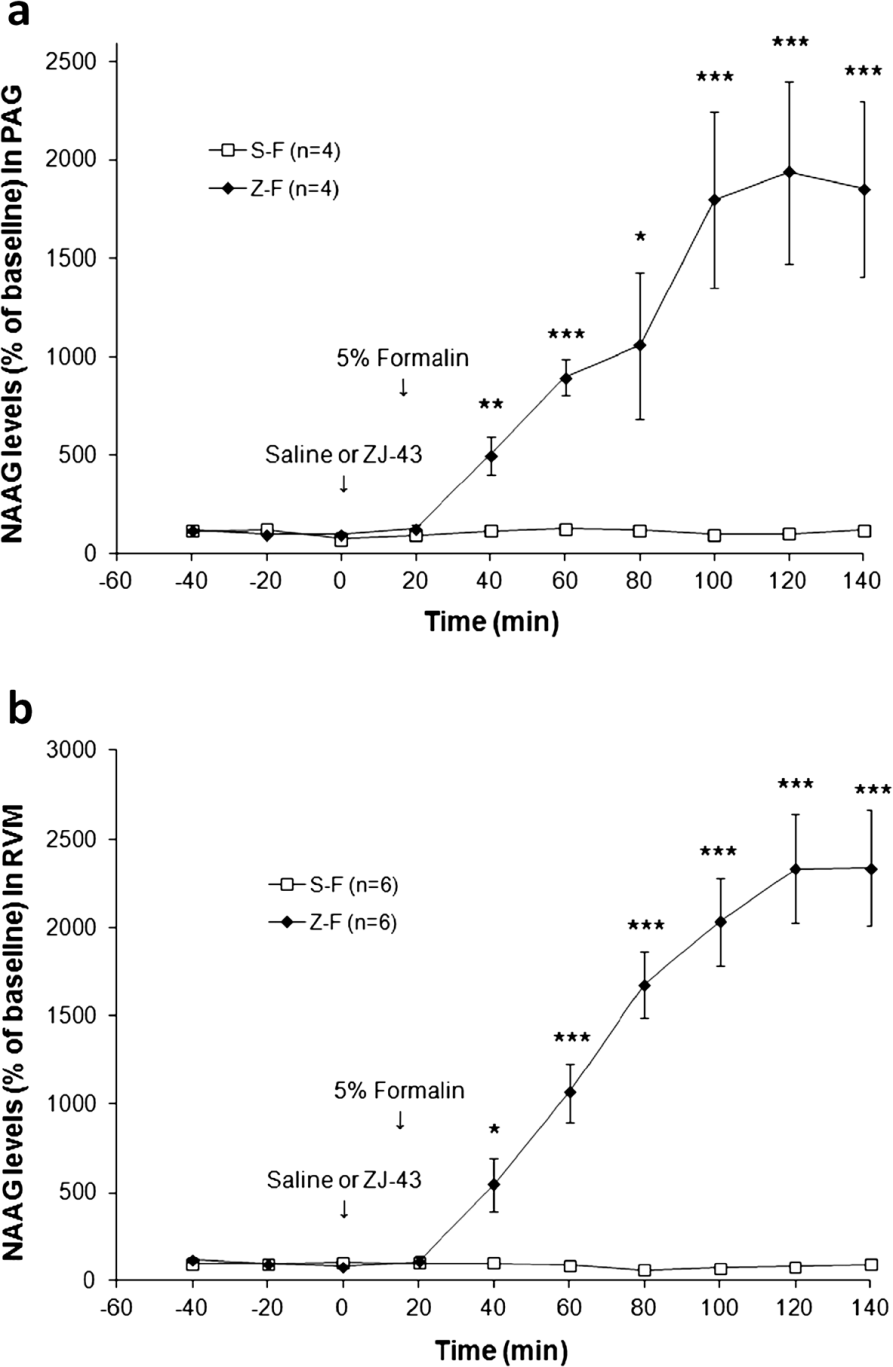
**Microdialysis sampling for NAAG release in the PAG (a) and RVM (b) following inflammation.** NAAG levels are expressed as a percent of the three baseline samples for each animal. ZJ43 (150 mg/kg, i.p.) prior to formalin treatment (Z-F) or saline prior to formalin (S-F). ZJ43 or saline was injected i.p. at 0 time followed by 50 μl of 5% formalin injected into the contralateral footpad at the 15 min time point. ZJ43 significantly (p < 0.001) increased NAAG levels in the PAG (**a**) and RVM (**b**). Samples were collected over 20 minute intervals. *p < 0.05; **p < 0.01; ***p < 0.001.

Formalin injection had no effect on NAAG levels in PAG and RVM (Figure 9a, b). ZJ43 increased NAAG levels by ~20-fold. There was a significant difference in NAAG levels between groups in the 0–140 min PAG dialysate samples (*p* < 0.01), and an overall time × group interaction was observed for NAAG in PAG (F_6, 36_ = 7.711, *p* < 0.001). There was a significant difference in NAAG levels between groups in the 0-140 min RVM dialysate sample (*p* < 0.001) and an overall time × group interaction was observed for NAAG in RVM (F_6, 60_ = 24.368, *p* < 0.001).

**Figure 10:**
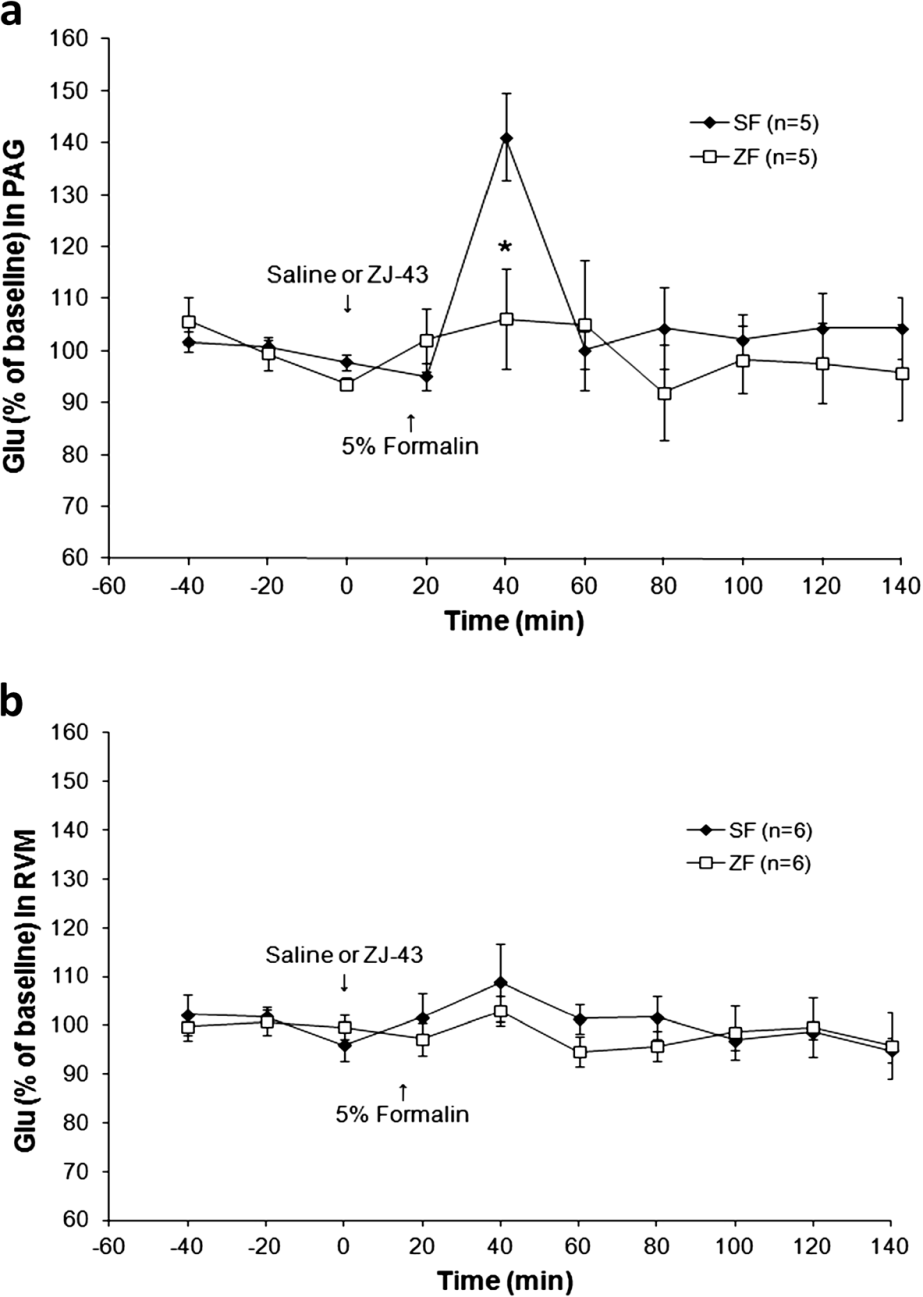
**Microdialysis sampling for glutamate release in the PAG and RVM following inflammation as in Figure**9**.** Glutamate levels are expressed as a percent of the three baseline samples for each animal. Saline (S-F) or ZJ43 (Z-F, 150 mg/kg, i.p.) was injected at 0 time, formalin at the 15 minute time point. **a**. Microdialysis in the PAG. Inflammation significantly increased (p < 0.05) glutamate levels between the 20- and 40-minute time points (5–25 minutes after formalin injection) in the PAG relative to baseline samples. ZJ43 blocked inflammation-induced glutamate levels relative to saline treatment at over this interval (p < 0.05). **b**. Microdialysis in the RVM. Formalin-induced changes in extracellular glutamate levels above baseline were not detected using the standard 2 mm dialysis tip following saline–formalin (S-F) or ZJ43-formalin Z-F) treatments using the standard 2 mm dialysis tip nor with a 1 mm dialysis tip (S-F-1 mm). *p < 0.05. The same microdialysates samples from the PAG and RVM were analyzed for both NAAG (Figure 10) and glutamate (Figure 9).

Formalin injection into the footpad resulted in a 40% increase in glutamate release over baseline in the contralateral PAG between 5 and 25 minutes after the inflammatory insult (Figure 10a). Systemic injection of ZJ43 (150 mg/kg, i.p.) blocked this increase in glutamate release as our model predicted. There was a significant difference in glutamate levels between groups in the 20–40 min dialysate sample (*p* < 0.05), and an overall time × group interaction also was observed for glutamate (F_2, 16_ = 5.57, *p* < 0.05). In the contralateral RVM, there was a small and not statistically significant increase in glutamate release in response to footpad inflammation and this was unaffected by systemic ZJ43 treatment (Figure 10b). The use of a smaller dialysis probe (1 mm vs 2 mm) similarly showed no effect of inflammation on glutamate levels in the RVM.

## Discussion

Current analgesic therapies typically inhibit cyclooxygenase enzymes or activate opiate receptors. Each of these front line analgesics for treatment of inflammatory and neuropathic pain has some negative clinical consequences or lacks efficacy in a significant proportion of patients. Adjuvant analgesics, including antidepressants (SSRIs), anticonvulsants (gabapentin, pregabalin) and anesthetics (mexiletine, lidocaine) are sometimes effective in relieving pain. However, they do not represent widely effective treatments for relief of chronic inflammatory or neuropathic pain or hyperalgesia.

The NAAG receptor, mGluR3, is widely expressed by neurons and glia in the nervous system including the PAG and RVM [34]. The observation of NAAG immunoreactivity (Figures 1a–c) in the ascending and descending pain pathway supports the potential of NAAG peptidase inhibitors to affect the response to inflammation.

The efficacy of NAAG peptidase inhibition in the PAG is consistent with the report that intra-PAG injection of the group II mGluR agonist L-CCG-I also reduces responses in the formalin model [35]. In previous studies, we found that peripheral [8], intrathecal [4] and intracerebroventricular [7] administration of NAAG peptidase inhibitors had similar analgesia-like properties in the formalin test. The data in this manuscript provide the first insight into the potential of NAAG and the inhibitors of its inactivation to influence the ascending (PAG) and descending (RVM and PAG) pain pathways. Given the expression of mGluR3 receptors and NAAG in the spinal cord and spinal sensory neurons [10,33], our previous studies and the data presented here suggest that, like opiate peptides, NAAG has the potential to moderate the response to inflammatory pain at several different levels within this pathway. The efficacy in this model of NAAG peptidase inhibition in the PAG and RVM support the conclusion that, at a minimum, the release of NAAG in these regions modulates the spinal pain response via the descending inhibitory pain pathway. This hypothesis needs to be further tested in other models that more clearly assess perception of pain since flinching in the formalin model can be critiqued as a spinal reflex that does not reflect cortically based cognition of pain. Relevant to this question, we previously reported that systemic and intrathecal injections of two NAAG peptidase inhibitors were highly effective in the sciatic nerve ligation and carrageenan pain models [5,6]. Additionally, we found NAAG peptidase inhibition to be analgesic in a model of bone cancer pain [29].

A series of studies demonstrate that NAAG peptidase inhibition elevates extracellular NAAG levels with the consequent activation of a group II mGluR (mGluR3), an activity that inhibits the release of small amine transmitters including glutamate, GABA, and aspartate, via presynaptic inhibition [16-20]. While other mechanisms of action are possible, presynaptic inhibition might well be responsible for the efficacy of NAAG peptidase inhibitors in animal models of several nervous system disorders [3].

Figure 10a presents the first demonstration of inflammation-induced increase in glutamate release in the PAG, a result consistent with increased ascending excitatory signals from the site of inflammation. Given the efficacy of ZJ43 microinjection into the PAG in reducing flinching and increasing extracellular NAAG levels, we examined the effect of NAAG peptidase inhibition on this inflammation-induced glutamate release. Systemically applied ZJ43 (50 mg/kg) reaches a concentration of 2 nM in the brain 30 minutes after injection [19] and significantly reduces NAAG hydrolysis in the rat brain *in vivo* (Olszewski et al., submitted). Its efficacy in blocking this inflammation-induced glutamate release in the PAG is consistent with our models of NAAG activation of presynaptic mGluR3 to inhibit transmitter release [3,33]. The difference between the RVM and PAG with respect to formalin-induced glutamate release could reflect the role of the PAG, but not the RVM, in the ascending pain pathway. While these data demonstrate a role for NAAG in the control of inflammation-induced glutamate release in the PAG, they are not sufficient to prove that the NAAG peptidase inhibition-mediated decrease in glutamate release mediates the observed reduction in the inflammation-induced motor response.

Microinjection of ZJ43 into the RVM also reduced the response to footpad inflammation (Figures 6,7) and systemic treatment with this inhibitor also elevated RVM NAAG levels (Figure 9b). In contrast to the PAG, however, inflammation did not significantly elevate glutamate levels in the RVM. Since microinjection of excitatory amino acids into the RVM is analgesic [36], it would not be expected that formalin treatment would necessarily produce a substantial increase in glutamate release or that inhibition of glutamate release in the RVM would mediate analgesia. One interpretation of these data is that NAAG activation of mGluR3 receptors inhibited the release of other transmitters in the RVM with the consequent effect on the local circuitry [37,38]. For example, inhibition of GABA release could indirectly result in an increase in release of other transmitters, whose actions mediate analgesia in the RVM [39,40]. Alternatively, the effect of formalin injection on glutamate release in the RVM might have been restricted to a volume of tissue that was smaller than that sampled by the microdialysis probe resulting in a failure to detect increases in glutamate levels above the background in the sampling area. However, a small study (n = 3) obtained using a smaller (1 mm) dialysis probe tip in sampling the RVM provided no evidence of an inflammation stimulated increase in glutamate release Figure 10b.

Heterotropic group II mGluR (mGluR2 and mGluR3) agonists reduce inflammatory pain responses and also may represent a novel analgesic strategy [1]. However, these compounds were tested in mGluR2 and mGluR3 knockout mice in animal models of schizophrenia and were found to be effective in mGluR3 but not mGluR2 knock outs [41,42]. In the same animal models, NAAG peptidase inhibition was effective in the mGluR2 but not the mGluR3 knockout mice [43]. These data support the conclusion that the heterotropic mGluR2/3 agonists and mGluR2 positive allosteric modulators have the potential to be effective mGluR2 based analgesic strategies in contrast to NAAG peptidase inhibition that represents an mGluR3 specific strategy. Also relevant to the differences between these two analgesic approaches, pharmacotherapies, such as antidepressants, sedatives and anxiolytics, that increase the activity of endogenous transmitters tend to enhance the normal ongoing physiology and thus can have less potential for secondary effects than continuous agonist-based receptor activation.

The concept that orally available NAAG peptidase inhibitors [2,26] might ultimately be used clinically for the treatment of inflammatory and neuropathic pain begs the question as to their potential secondary effects inasmuch as the peptide and mGluR3 are widely distributed in the nervous system. Studies in mice do not suggest that negative secondary effects result from NAAG peptidase inhibition [33]. For example, we found no significant neurological deficits in mice in which the major NAAG peptidase, glutamate carboxypeptidase II, had been knocked out [44]. Similarly, chronic treatment with a NAAG peptidase inhibitor was without detectable side effects in a study where the drug increased the lifespan of mice in a model of amyotrophic lateral sclerosis [45]. Acute treatment with ZJ43 similarly lacks detectable effects in open field behavior [20], prepulse inhibition of acoustic startle [46], or the 1.5 hr delay novel object recognition test [Olszewski et al., submitted]. Neither systemic NAAG peptidase inhibition [6] nor microinjection of 150μg of ZJ43 into the PAG and RVM (Figure 8) alter the reaction time of rats in the hot plate test. This lack of apparent side effects is consistent with the established pattern of peptide co-transmitter release under conditions of high neuronal activity with little released during normal to low levels of activity. Consistent with this concept, the basal extracellular concentrations of NAAG and glutamate in the dialysates, uncorrected for recovery, were 53 ± 06 nM and 1,250 ± 130 nM in PAG, 74 ± 5 nM and 820 ± 70 nM in RVM respectively. This difference in extracellular concentrations also is found in other brain regions [19,20] despite the fact that NAAG is present in mM concentrations in the mammalian nervous system [47]. Such a pattern of release is consistent with our model of NAAG feeding back on presynaptic mGluR3 to dampen synaptic release of primary amine transmitters under conditions of high activity [3].

## Conclusions

The data presented here provide the first demonstration of: 1) the presence of NAAG within neurons and presumptive synaptic endings in two discrete regions of the pain processing pathway of the brain, the PAG and RVM; 2) the elevation of synaptically released NAAG in these sites by systemic application of a NAAG peptidase inhibitor; 3) the analgesic efficacy of NAAG peptidase inhibition directly in the PAG and RVM; 4) the role of the mGluR3 receptor in mediating the analgesic efficacy NAAG peptidase inhibition in the PAG and RVM based on NAAG’s selectivity for this receptor [11] and the blockade of the effect NAAG peptidase inhibition by co-administration of the mGluR2/3 antagonist LY431495. These data support the conclusion that NAAG peptidase inhibition has potential as an approach to treating inflammatory pain via either the ascending (PAG) and/or the descending pain pathways (PAG and RVM) and warrants further study.

## Materials and methods

The Institutional Animal Care and Use Committees at Georgetown and Kumamoto University approved all animal procedures. The principles of animal care were consistent with the standards of the US National Institutes of Health and Department of Agriculture. Male Sprague–Dawley rats (300–350 g) were housed in groups of two, maintained on a 12-hour dark–light cycle, and permitted food and water *ad libitum*. Animals were handled on arrival and were housed for at least three days before testing. Behavioral testing was performed between 10.00 h and 16.00 h. Animals were euthanized immediately after behavioral or microdialysis studies.

### Immunohistochemistry and antibodies

The NAAG specific antisera were prepared and immunohistochemistry performed as previously described [48,49]. Briefly, polyclonal NAAG antisera were purified in stages by both affinity chromatography and negative-affinity adsorption against related protein-coupled molecules including N-acetylaspartate N-acetylglutamate glutamate, aspartate and GABA [48]. Rats were transcardially perfused with 6% carbodiimide and 5% DMSO. Brains were post fixed with 4% paraformaldehyde, saturated with 30% sucrose, frozen and sectioned (20 μm). Sections were treated with 2% normal goat serum prior to incubation with affinity purified anti-NAAG rabbit serum (1:2,000). Antibodies were visualized with peroxidase-labeled avidin-biotin complex (Vectastain, Vector Labs, Burlingame, CA) and developed with H_2_O_2_ as substrate for diaminobenzidine as chromogen. Control tissue sections lacking antibody treatment or treated with NAAG blocked antibody exhibited no specific reaction product above background. These antibodies fail to cross react with glutamate, N-acetylaspartate, N-acetylglutamate, aspartate or GABA [48,49]. Images were acquired on an Olympus BX51 microscope and DP71 camera, and were adjusted for contrast and brightness using PC based software (Adobe Systems). Additional software was used for enhanced depth of field by taking multiple images at different focal points in a tissue slice, and combining them into a single focused image (Media Cybernetics).

### PAG and RVM cannulae placement for microinjection

Implantation of the injection cannulae into the PAG and RVM was performed under halothane anesthesia. The rats were placed in a stereotaxic apparatus (KOPF Model 900, David Kopf Instruments, CA) and stainless steel 26 G thin wall guide cannulae (C315G, PlasticsOne, One, Roanoke, VA) were stereotaxically placed at either PAG through a burr hole (AP: -7.6 mm, L: 0.7 mm, H: 6.0 mm from Bregma) or RVM through a burr hole (AP: 11.0 mm, L: 0.0 mm, H: 11.0 mm from Bregma). Guide cannulae were affixed to the skull with stainless steel screws and cranioplastic cement. 32.5 mg mefenamic acid (Daiichi-Sankyo, Tokyo, Japan) was orally administered 2 times/day at 0 – 2 days after surgery for postoperative pain control. The formalin inflammation tests were performed 7 days after cannulae implantation. All animals displayed normal feeding and drinking behaviors post-operatively. Rats showing neurological deficits after cannulae implantation were not studied.

### Drugs and microinjection

The NAAG peptidase inhibitor ZJ43 (IC_50_ = 2.4 nM, Ki = 0.79 nM for human glutamate carboxypeptidase II [28]) was synthesized following methods previously described [28] and provided by Alan Kozikowski. LY341495, a highly selective group II metabotropic glutamate receptor antagonist [50], was purchased from Tocris and injected i.p. (1 mg/kg) in a total volume of 1 ml. ZJ43 and LY341495 were dissolved in saline. For the PAG and RVM microinjections, ZJ43 or saline was delivered in a total volume of 0.5 μl over a period of 60 seconds using a microsyringe pump (EP-60, Eicom) and a 30 G stainless steel internal cannula (C315G, Plastics One) connected via a polyethylene tube to a 10 μl Hamilton syringe.

At the completion of the experiment, 0.5 μl of India ink was injected into the PAG or RVM 10 min before rats were euthanized. The brains were fixed with formalin, and coronal tissue sections were Nissl stained to confirm the proper injection site. Only the rats whose microinjection site was located within PAG or RVM were included in the results.

### Footpad inflammation test

In the formalin test, 50 μl of 5% formalin was injected subcutaneously (SC) into the dorsal surface of the right hind paw with a 25-gauge needle under brief halothane anesthesia. Within 1 min after the formalin injection, spontaneous flinching of the injected paw could be observed. Flinching is readily discriminated and is characterized as a rapid and brief withdrawal or flexion of the injected paw. This pain-related behavior was quantified by counting the number of flinches for 1 min periods at 1–2 and at 5–6 min, and then for 1 min periods at 5 min intervals from 10 to 60 min after the injection. Two phases of spontaneous flinching behavior were observed: an initial acute phase (phase 1: during the first 6 min after the formalin injection) and a prolonged tonic phase (phase 2: beginning about 10 min after the formalin injection).

For the dose–response studies, ZJ43 was microinjected into the PAG or RVM 10 min before the formalin injection into the footpad. In the PAG studies, the peptidase inhibitor was tested separately contralateral and ipsilateral to the injected footpad. For the PAG, 150 μg treated group: n = 7; 37.5 μg treated group: n = 5; 3.75 μg treated group: n = 6; saline: n = 6. To verify whether the effect of ZJ43 injected to PAG contralateral to the formalin injection on the formalin test was mediated by the action of ZJ43 on the site of injection, 150 μg (n = 6) of ZJ43 was administered to the PAG ipsilateral to the formalin injection 10 min before the formalin injection. For the RVM, 150 μg treated group: n = 7; 15 μg treated group: n = 5; 1.5 μg treated group: n = 5; 0.15 μg treated group: n = 5; saline: n = 7. To verify that the analgesic effect of ZJ43 in the PAG and RVM was mediated by the activation mGluR3, 1 mg/kg of LY341495 was administered (i.p.) 10 min before the microinjection of ZJ43 (150 μg) into the PAG (n = 7) or RVM (n = 10).

### Hot plate test

Rats were placed on a 52.5°C surface. The response latency to either a hind-paw lick or a jump was recorded. In the absence of a response, animals were removed from the 52.5°C hot plate at 60 sec (cut-off time) and a 60 sec latency was assigned as the response.

Three baseline measurements were made before the drug injection. ZJ43 (150 μg) was administered into PAG (n = 4) or RVM (n = 4) and the hot-plate latency was measured at 5, 15, 30, 45 and 60 min after the drug injection. To obtain control data, the vehicle was injected into PAG (n = 6) or RVM (n = 4).

### Microdialysis and assay of glutamate and NAAG levels

Rats were anesthetized with Ketamine (80 mg/kg)/Xylazine (5 mg/kg) (i.p.) and placed in a stereotaxic apparatus (KOPF Inc., Tujunga, CA, USA) for surgical implantation of a guide cannula. The guide cannula (SciPro Inc., Sanborn, NY, USA) was positioned to the (contralateral) PAG according to the coordinates (AP, - 7.8; ML, -0.7; DV, -4.4) or RVM according to the coordinates (AP, - 11.0; ML, -0.7; DV, -9.0). The guide cannula was secured to the skull with dental cement anchored by two stainless steel screws. After surgery, each rat was individually housed and allowed to recover for 24 hours before microdialysis and footpad injection.

Microdialysis was carried out on conscious, freely moving rats. Rats were lightly anesthetized with isofluorane to facilitate manual insertion of the microdialysis probe into the guide cannula. The stylet in the guide cannula was replaced with the microdialysis probe (outer diameter, 0.6 mm; exposed tip, 2.0 mm; cut-off of 6 kDa; SciPro Inc., Sanborn, NY, USA). The rats were tethered to the awake animal system by means of a plastic collar (CMA Microdialysis AB, Sweden). The probe was perfused at 2 μl/min with artificial cerebrospinal fluid (CMA Microdialysis AB, Sweden). After at least 2 h to reach equilibration, dialysate samples were collected every 20 min. Three baseline fractions were collected before ZJ43 or saline injection. Pretreatment of ZJ43 (150 mg/kg i.p.) or saline occurred 15 min before injecting formalin (5%, 50 μl) into the dorsal side of the left hind-paw during microdialysis. Samples were subsequently analyzed for NAAG and glutamate. At the completion of each experiment, rats were euthanized and brains were removed, fixed in 10% formalin and 30 μm coronal sections prepared to confirm probe placement.

### NAAG and glutamate analysis

Glutamate in the microdialysate samples was derivatized with o-phthalaldehyde (OPA, Sigma, USA) and resolved by reverse phase HPLC (Atlantis T3, ODS, 4.6 × 150 mm, 3 μm) and electrochemical detection (Waters). Quantification was obtained from standard curves prepared over a glutamate range of 0.1–100 μM. Dialysate samples were analyzed for NAAG content using radioimmunoassay as previously described [48] with minor modifications. Briefly, 20 μl samples diluted to 50 μl with PBS were incubated overnight at 4°C with 25 μl of NAAG antisera (1:25) and 25 μl of ^3^ H]-NAAG (50,000 cpm, approximately 5 pmol). After incubation, 900 μl of −20°C methanol was added, precipitated proteins were separated by sedimentation at 15,000 x g for 15 min and tritium in the pellet and supernatant was quantified. ^3^ H]-NAAG bound to antibody in unknown samples was compared to that of a standard curve (0.033–3.3 μM NAAG) that was repeated with each assay.

### Statistical analysis

The time-response data are presented as the mean flinches (± SEM) per minute for the periods of 1–2 min and 5–6 min and then for 1 min periods at 5 min intervals up to 60 min. For the dose–response analysis, data from phase 1 (0–2 min) and phase 2 (10–60 min) observations were considered separately. In each case, the cumulative instances of formalin-evoked flinches during the phase 1 and phase 2 were calculated for each rat. These individual rat data were then used to construct phase 1 and phase 2 dose–response curves. For dose–response data and tests of the efficacy of LY341495, one-way ANOVA was used with Tukey post-hoc test.

For the analysis of drug effect, the % maximum possible effect was calculated, where % maximum possible effect = ([post-drug maximum latency – pre-drug latency]/[cut-off time (60 sec) – pre-drug latency]) x 100. The post drug maximum latency was defined as the single longest latency during the entire time course of the hot plate test. To analyze the effect of ZJ43 on % maximum possible effect, an unpaired *t*-test was used.

Microdialysis data are expressed as percentage of basal values (calculated as means of the three samples before injections). Data on the responses following ZJ43 injection in the ipsilateral versus contralateral PAG were compared using Students *t*-test. The basal concentrations of NAAG and glutamate in the dialysates, uncorrected for recovery, were 0.053 ± 0.006 μM and 1.25 ± 0.13 μM in PAG, 0.074 ± 0.005 μM and 0.82 ± 0.07 μM in RVM respectively. The values were expressed as a % of baseline level for each rat and the mean and standard error were determined for each treatment group. Two-factor ANOVA for repeated measurements was used to examine the possibility of significant differences (p <0.05) between groups followed by Tukey post hoc test.

Microdialysis data are expressed as percentage of basal values calculated independently for each animal as mean of the three samples taken before injections. All data were given as mean ± standard error of the mean (S.E.M.) and not corrected for ‘recovery’ of the dialysis procedure. General Linear Model with repeated measurements (SPSS19 for Windows) was used to examine the possibility of significant differences (p < 0.05) between groups followed by a Student’s *t*-test for every time-point.

## Abbreviations

ZJ43: Is a NAAG peptidase Inhibitor; mGluR3: Is the NAAG receptor (metabotropic glutamate receptor 3); LY341495: Is an mGluR2/3 agonist; PAG: Is periaqueductal gray; RVM: Is Rostral Ventromedial Medulla; RM: Is raphe magnus.

## Competing interests

Georgetown University holds the patent rights to ZJ43. The authors have no interests in this patent. Authors have no commercial associations that might pose a conflict of interest in connection with the submitted article.

## Authors’ contributions

JM performed immunohistochemistry; JN designed the research and wrote the manuscript; TY designed and directed the research; TY executed the microinjection studies; DZ executed the microdialysis studies; RO assisted with the RIA of NAAG, assembled and edited the manuscript; TB assisted with the HPLC and analysis of glutamate and dopamine, edited the manuscript. All authors read and approved the final manuscript.
